# Multiparametric Analysis of Circulating Exosomes and Other Small Extracellular Vesicles by Advanced Imaging Flow Cytometry

**DOI:** 10.3389/fimmu.2018.01583

**Published:** 2018-07-06

**Authors:** Sotiris Mastoridis, Giuliana Minani Bertolino, Gavin Whitehouse, Francesco Dazzi, Alberto Sanchez-Fueyo, Marc Martinez-Llordella

**Affiliations:** ^1^MRC Centre for Transplantation, Institute of Liver Studies, King’s College London, London, United Kingdom; ^2^Regenerative Medicine, Division of Cancer Studies and Cancer Research UK King’s Health Partners, King’s College London, London, United Kingdom

**Keywords:** exosome, vesicle, cytometry, tetraspanin, imageStream

## Abstract

Extracellular vesicles (EVs) are responsible for a multitude of physiological functions, including immunomodulation. A heterogenous mixture of small EV (sEV) subsets, including putative exosomes, is derived when commonly used “exosome” isolation techniques are employed. Subset diversity relates in part to their different intracellular origins, and can be associated with distinct functional properties. Recent progress in the EV field has enabled the categorization of such subsets based on their surface composition. For the first time, we combine such emerging subset-specific markers with advanced imaging flow cytometry (iFCM) to perform high-throughput, multiparametric, vesicle-by-vesicle characterization, and functional assessment of specific small EV subsets, and exosomes in particular. The approach allows researchers to address three important applications. First, it is known that different isolation techniques result in the divergent recovery of particular vesicle subsets. Taking three commonly used “exosome” isolation techniques as test cases (ultracentrifugation, size-exclusion chromatography, and polymer-based precipitation), the capacity for convenient and accurate isolate compositional analysis by iFCM is demonstrated. The approach was able to corroborate and to quantify the known skewing of subtype recovery among different isolation approaches. Second, exosomes are a particularly widely studied EV subset. Applying exosome-specific markers to samples collected from an optimal clinical transplantation model, we verify the capacity for iFCM to detect exosomes in circulation, to establish their tissue of origin, and to provide insights as to their functional immunological potential. Finally, we describe a technique for establishing whether the transfer of a molecule of interest to a target cell is exosomally mediated. In so doing, we highlight the approach’s utility in assessing the functional *impact* of circulating exosomes and in identifying their targets. In conclusion, we set out a new methodological approach by which small extracellular vesicle subsets, exosomes in particular, can be conveniently and comprehensively investigated, thereby offering novel phenotypic and functional insights.

## Introduction

Extracellular vesicles (EVs) are nanosized membrane-bound particles released by most cell subsets. Owing to their capacity to transport a variety of biomolecules, they are key mediators of intercellular communication and as such, are the focus of increasing research interest (1–3). Much of this work has brought to light the capacity for EVs to regulate the immune system, with EVs from both immune and non-immune cells shown to be able to stimulate or suppress innate and adaptive immune responses (1, 3–5). Among their surface protein cargo, EVs can carry MHC and peptide/MHC complexes, costimulatory molecules, and adhesion molecules. The extracellular domains of these remain exposed on the vesicle surface thereby preserving their functionality, with their topology mirroring to an extent that of the parent cell (6).

As a field in its infancy, the characterization and classification of subtypes of EVs remain in flux. It is increasingly evident that diverse subpopulations of EVs secreted by different intracellular mechanisms and displaying varying functional properties exist. Such heterogeneity holds true for EVs in the 50–150 nm size range, often collectively referred to as exosomes, but more accurately described as “small EVs” (sEVs)—a term which is coming into more widespread use (7, 8). That is to say, isolates previously assumed to contain “exosomes” are now recognized to contain a mixture of exosomal and non-exosomal sEVs. Exosomes are defined in part by their small size, but also by their content of endosome-associated proteins relating to their unique endosomal biogenesis. The tetraspanin protein CD63 is particularly associated with endosome-derived exosomes. sEVs enriched in CD9 but lacking CD63 and CD81 are associated with an early endocytic signature and the plasma membrane. Finally, sEVs not associated with the endosomal pathway were devoid of all three of these tetraspanin markers (7). Further to these insights, recent observations indicate that variations in sEV tetraspanin content in fact denotes different functional properties since, as well as playing an important role in sEV biogenesis, tetraspanins regulate the routing and sorting of specific biomolecules into sEVs (9–11).

A convenient approach to vesicle-by-vesicle analysis employing such markers has, however, been lacking, making subset-specific analyses difficult. Such analysis of individual sEVs is primarily restricted by their size. Techniques which permit their visualization, such as electron microscopy or atomic force microscopy, preclude the analysis of sEVs in large numbers, thereby limiting robust statistical assessments of heterogeneity. Proteomics, Western blotting, lipidomics, and flow cytometry of bead-captured vesicles are useful methods in the analysis of bulk isolates but are unable to distinguish variations in the number of vesicles from changes in molecular composition and are incapable of multiparametric analysis of single sEVs. Although flow cytometry is an ideal technique for high-throughput and multiparametric characterization of individual cells, most conventional flow cytometry (cFCM) platforms suffer from detection thresholds above 500 nm (12–15). With more than 80% of EVs reported to be <500 nm in size, the majority of cFCM analyses of EVs to date have characterized only the tip of the EV iceberg, with the smallest EVs including exosomes remaining particularly elusive (16).

Advanced imaging flow cytometry (iFCM) by ImageStream^x^ (ISx, EMD Millipore, Seattle, WA, USA) has been shown to offer significant advantages over cFCM in this regard (14, 17). The capacity for submicron particle and small EV (sEV) detection by iFCM has been comprehensively validated by Erdbrugger and colleagues in recent publications (14, 18). Here, we demonstrate a novel approach combining iFCM with subset-specific markers for the high-throughput, multiparametric, characterization, and functional assessment of circulating exosomes in particular, as well as other sEV subsets. A methodological framework for such analysis is provided, and we set out three key applications of this approach in order to assist researchers in the robust subset analysis of heterogeneous sEV isolates, and for the sensitive detection, phenotyping, and functional impact assessment of bona fide circulating exosomes.

## Materials and Methods

### Preparation of Blood Plasma

All sample collection and preparation protocols were approved by the North of Scotland Research Ethics Committee (REC Ref: 15/NS/0062). Participants provided written informed consent. Peripheral blood was collected following standard procedures that minimize contamination by platelets and platelet-derived vesicles (19). Briefly, following cubital vein venepuncture, 3 mL of blood was discarded before collection of 9 mL into BD Vacutainer^®^ K3-EDTA-coated collection tubes (Beckton Dickinson, USA). Tubes were inverted gently five times and blood was allowed to sit at room temperature for 30 min. Whole blood was then centrifuged (Heraeus Megafuge 40R with 195 mm 7500-3180 rotor, Thermo Scientific) at 400 *g* for 10 min at 20°C to remove cells. The plasma layer was collected and centrifuged again at 5,000 *g* for 10 min at 20°C. The resulting platelet poor plasma (PPP) was aliquoted and stored at −80°C.

### sEV Isolation

Small EVs were isolated from PPP by three commonly used “exosome” isolation techniques—ultracentrifugation (UC), size-exclusion chromatography (SEC), and polymer-based precipitation (PBP) (20–22). For UC, PPP was centrifuged (Sorval Legend Micro 21R equipped with 7500-3424 rotor, Thermo Scientific) at 10,000 *g* for 30 min. 0.85 mL supernatant was resuspended in 10 mL of 0.22 µm-filtered (Merck) phosphate-buffered saline (fPBS) in ultracentrifuge tubes (Ultra-Clear Tube, Beckman Coulter), and putative exosomes (sEVs) were pelleted at 100,000 *g* for 70 min at 4°C in a SW41 Ti rotor (Beckman Coulter). The pellet was resuspended in 200 µL fPBS. SEC was performed using CellGS Exo-Spin™ Mini Columns according to manufacturer’s instructions. In brief, PPP was centrifuged at 16,000 g for 30 min (Sorval Legend Micro 21R equipped with 7500-3424 rotor, Thermo Scientific). Following equilibration of columns with 200 µL fPBS centrifuged for 10 s at 50 *g* (Centrifuge 5430R, equipped with FA-45-24-11-HS rotor, Eppendorf), 0.1 mL PPP was applied to the column and centrifuged at 50 *g* for 60 s. The column was then transferred to a new 1.5 mL collection tube, 200 µL fPBS applied to the top, and elution of putative exosomes (sEVs) performed by a final centrifugation step at 50 *g* for 60 s. Eluate volume was topped up to 200 µL if necessary with fPBS, aliquoted, and stored at −80°C. PBP was performed by ExoQuick™ (System Biosciences, Mountain View, CA, USA) according to manufacturer’s instructions with minor modifications. In brief, PPP was treated with 0.5 U/mL thrombin at room temperature for 5 min, then centrifuged at 10,000 *g* for 5 min (Sorval Legend Micro 21R equipped with 7500-3424 rotor, Thermo Scientific). The 0.25 mL supernatant was incubated for 30 min at 4°C with 63 µL Exoquick solution, and then centrifuged at 1,500 *g* for 30 min. The putative sEV/“exosome” pellet was then resuspended in 200 µL fPBS, aliquoted, and stored at −80°C.

### Nanoparticle Tracking Analysis (NTA)

Size distribution and concentration of isolated vesicles were measured by NanoSight NTA (LM10, Malvern Inst. Ltd., UK). The NTA analyses the motion of particles illuminated by a laser, from which it deduces their size and concentration. Samples were diluted (1:5,000) with fPBS and readings taken in triplicates over 30 s, with manual monitoring of temperature and camera level set to 14. Analysis, including taking averages of triplicate readings, was performed using NTA v3.1 software, with detection threshold set to 7.

### Protein Estimation and Western Blot

Small EV preparations were diluted (1:2) in lysis buffer with final concentration of 50 mM Tris-Cl (Promega, Medison, USA), 150 mM NaCl (BDH, Poole, UK), 1% v/v Triton™ X-100 (BDH), 0.5% v/v sodium deoxycholate (Sigma-Aldrich, St. Louis, USA), 3% v/v sodium dodecyl sulfate (Merk, San Diego, CA, USA) and in presence of 1 mM phenylmethylsulfonyl fluoride (Sigma-Aldrich), Complete Protease Inhibitor Cocktail (Cat #04693124001, Roche, Mannheim, Germany), and Halt Phosphatase Inhibitor Cocktail (Cat #04906837001, Roche). Samples were solubilized on ice for 30 min, followed by centrifugation at 13,000 *g* for 15 min at 4°C. Supernatant was collected and protein estimation was performed using Novagen BCA kit (Merk) as per manufacture’s protocol. Twenty micrograms of protein lysate was diluted in 2× sample buffer [4% sodium dodecyl sulfate, 20% glycerol (Sigma-Aldrich), 0.004% bromphenol blue (Sigma-Aldrich), and 0.125 M Tris-Cl pH 6.8] to a final volume of 30 µL and loaded into 10% SDS-PAGE gel in non-reducing conditions. The electrophoresis was performed with continuous buffer system and proteins were transferred by wet electroblotting onto polyvinylidene difluoride (PVDF) membranes (Bio-Rad, Hercules, CA, USA). To confirm the transfer and validate that all samples contained similar amounts of protein, the PVDF membrane was stained for 5 min with ATX Ponceau S red staining solution (Sigma-Aldrich). Membranes were blocked in 5% non-fat milk (Bio-Rad) in Tris-buffered saline containing 0.05% Tween-20 (Sigma) for 1 h, and then probed with the following primary antibodies: anti-CD63 (TS63, 1:1,000, Life Technologies, Carlsbad, CA, USA), anti-CD9 (M-L13, 1:250, BD Biosciences, San Diego, CA, USA), and CD81 (M38, 1:500, Life technologies) for 18 h at 4°C. Membranes were incubated with anti-mouse HRP-conjugated secondary antibody (Cat #P0260, 1:1,000, Dako, Glostrup, Denmark) for 1 h at room temperature. The proteins bands were visualized after incubation with Pierce ECL substrate (Thermo Scientific, Rockford, IL, USA) and development in Amersham Hyperfilm ECL (GE Healthcare, Buckinghamshire, UK).

### Transmission Electron Microscopy (TEM)

Small EV preparations were placed on Formvar-coated copper grids and allowed to settle for 5 min, without being allowed to dry. sEVs were then fixed with 2% glutaraldehyde for 5 min and subsequently briefly washed three times with distilled de-ionized water. After washing, the grids were stained for 20 min with 3% uranyl acetate: 2% methyl cellulose (1:9). Imaging of sEVs was carried out using a FEI Tecnai G^2^ transmission electron microscope, operated at 200 kV, fitted with a Gatan Ultrascan US1000 camera.

### EV Labeling and Small Particle Calibrators

Labeling was performed as previously described with minor adaptations (17, 18, 23). The ISx requires small volumes of EV sample (25–100 µL). In most instances we opted for 30 µL, adjusting buffer/reagent/antibody concentrations appropriately, since no wash steps are required or performed following labeling. The primary purpose of dilution is to find a balance between timely acquisition and potential complications associated with too high concentration—for instance “swarm.” ISx is less likely to suffer from such coincident detection than cFCM platforms, particularly if sample concentrations <10^10^ events/mL are used. 3 µL PPP, SEC, and PBP or 4.8 µL UC samples were topped up to 22 µL fPBS, and 0.5 µL Fc receptor blocker (Human TruStain FcX™, BioLegend) was added for 10 min at room temperature. 5-(and-6-)-carboxyfluorescein diacetate succinimidyl ester (CFDA-SE, ThermoFisher) was added to the EV-PBS solution to give a final running concentration of 10 µM (2.5 µL volume), allowed to incubate in the dark for 10 min at 4°C, and followed by further staining in the dark at room temperature for 15 min with master-mix preparations in fPBS (5 µL volume) of the following monoclonal antibodies (mAbs) as appropriate: anti-human HLA-A3-APC (eBioscience, GAP.A3); CD9-PE (BioLegend, HI9a); anti-human CD63-PE (BioLegend, H5C6); CD81-PE (BioLegend, 5A6); HLADR-PECY7 (Biolegend, L243), HLAB8-APC (Miltenyi, REA145), HLAB7 (Miltenyi, REA176); PD-L1-BV605 (Biolegend, 29E.2A3); IgG1 (BioLegend, MOPC-21); and IgG2b (BioLegend, MPC-11). All mAbs were centrifuged at 5,000 *g* for 5 min prior to use, as clumps could be mistaken for EVs (13, 18, 24). CFDA-SE is cleaved of acetate groups by EV esterases and converted to CFSE (carboxyfluorescein succiminidyl ester), which serves as a fluorescent pan-EV label. Optimized protocols for the labeling of EVs by such dyes have been published, and are preferred for their relative ease, specificity, and the circumvention of wash steps (18, 23, 24). To avoid false positive events, all antibodies used were run on ISx in buffer (fPBS) alone to ensure antibody clumps were not present. Verification of small particle detection was performed using fluorescently labeled submicron polystyrene beads (PSB) and liposomes. ApogeeMix (Apogee Flow Systems, UK) contained green beads 110 and 500 nm in size. Liposomes were prepared as previously described, with CF™-labeling performed by the addition of 1% 1,2-dioleoyl-sn-glycero-3-phosphoethanolamine-CF™488 (CF-DOPE) to the lipid film (25). EV lysis was performed by incubating fPBS-diluted EVs in 0.1% Triton™ X-100 (Thermo Scientific) for 15 min at room temperature.

### ImageStream^x^ Small Particle Acquisition and Analysis

Multispectral imaging flow-cytometric acquisition of EVs and small particle calibrators was performed using Amins ImageStream^x^ *MKII* (ISx, EMD Millipore, Seattle, WA, USA) with fluidics set at low speed, sensitivity set to high, magnification at 60×, core size 7 µm, and the “Hide Beads” option unchecked prior to every acquisition in order to visualize speed beads in analyses (Figure S1 in Supplementary Material). All parameters are stored in acquisition template except the latter, which requires unchecking prior to each acquisition. The ISx was equipped with the following lasers run at maximal power to ensure maximal sensitivity: 405 nm (120 mW), 488 nm (200 mW), 561 nm (200 mW), and 642 nm (150 mW). Upon each startup, the instrument calibration tool ASSIST^®^ was performed to optimize performance and consistency. Each of the two charged couple device (CCD) cameras with which the ISx is equipped have six channels of detection. Two channels (Ch01 and Ch09) were set to brightfield (BF), permitting spatial coordination between cameras. Channel 12 was set to side-scatter (SSC), and further fluorescence channels were used for antibody detection as required (Figure S1 in Supplementary Material). The advanced fluidic control of ISx, coupled with the presence of continuously running speed beads enable cell/particle enumeration using the “objects per mL” feature within the IDEAS^®^ data analysis software. To avoid the risk of coincident particle detection, EV samples were not run at concentrations greater than 10^10^ objects/mL (18). All samples were acquired using INSPIRE^®^ software, with a minimum of 5,000 G1 events collected, or as dictated by the type of analysis to be undertaken. Data analyses were performed, and spectral compensation matrices produced, using ISx Data Exploration and Analysis Software (IDEAS^®^) (Figure S2 in Supplementary Material). Technical controls and isotype controls, in conjunction with fluorescence minus one (FMO) controls, were employed where appropriate for EV gating.

### sEV Uptake Assay and ImageStream^x^ Analysis

Peripheral blood mononuclear cell isolation was performed in PBS-diluted (1:2) fresh blood collected in 3–4 6 mL collection tubes (Vacuette^®^, 456088) by Ficoll-Paque (GE Healthcare, Sweden) density-gradient centrifugation. Monocytes were separated by immunomagnetic beads coated with anti-CD14 antibodies according to EasySep™ manufacturer’s instructions (EasySep™, StemCell Technologies), yielding purities >90% as assessed by cFCM (Figure S3A in Supplementary Material) using (Heraeus Megafuge 40R with 195 mm 7500-3180 rotor, Thermo Scientific) at 800 *g* for 20 min with brake set to setting 3. Monocytes were cultured in 96-well plate (200,000/well) in serum-free media (X-VIVO™ 15, Lonza), with or without PBP-derived sEV added on a volume/volume basis to mirror physiologic conditions and circumvent errors intrinsic to sEV protein estimation (9). PBP isolation was performed on 500 µL PPP according to manufacturer’s instructions, with the final sEV pellet resuspended in 250 µL fPBS. 500 μl PPP is derived from approximately 1 mL fresh blood, and since each culture condition was performed in 200 µL, a fifth of the PBP isolate was utilized in each test condition. Summarily, monocytes were cultured either in 200 µL X-VIVO™ alone, or in 150 µL X-VIVO™ with 50 µL PBP isolate. Following incubation, adherent cells were collected by gentle pipetting following washing with ice cold fPBS and allowing to stand on ice with cold fPBS for 5 min. Cells were then labeled with the following antibodies: CD45-V450 (BD, H130); CD63-PECy7 (BioLegend, H5C6); HLA-A3-APC (eBioscience, GAP.A3); and LIVE/DEAD™-FITC (ThermoFisher Scientific). Whole-cell image acquisition was performed with fluidics set at low speed, sensitivity set high, magnification at 60×, and SSC and BF channels set as above. Live cells were identified after sequential gating for (i) single cells, using BF aspect ratio Vs. area feature gating; (ii) in focus cells, using BF gradient root mean square feature; and (iii) LIVE/DEAD™-stain negative cells (Ch02). Colocalization analyses were performed on further sub-gated HLA-A3-stain positive (Ch11) populations.

The two fundamental principles needed to understand the data analysis framework of IDEAS^®^ are Masks and Features (Figure S2 in Supplementary Material). Masks are used to spatially discriminate the area of the cell that is of interest and exclude those parts that are not. They can be created based on brightfield (BF), scatter (SSC), or fluorescence images. For instance, the morphology mask might be used for defining the nucleus, the spot mask for defining labeled cellular components by the identifying bright or dark regions, and so on. There are in the region of 20 masks available, and these can be combined in a Boolean manner to create multiple potential applications. Mask validation should always be performed by visually inspecting the mask on numerous acquired cells, and alternative approaches can often be found. Features on the other hand, are a computational algorithm able to analyze masks as their input. For instance, the quantitative assessment of internalization might be achieved by combining a spot mask from one detection channel with a morphology mask from another. The gamut of application potential and protocol descriptions have been comprehensively outlined elsewhere (26, 27). The approach to protein colocalization by ISx and its software package IDEAS^®^ is well described (28–30). In summary, ISx enables quantitative analysis of the degree of colocalization between fluorophores on a pixel-by-pixel basis by comparing digital images captured in each of its image detection channels. The Similarity Bright Detail R3 algorithm within IDEAS^®^ produces a score (SBDS) serving as a measure of the degree of colocalization between these. Comparison of the SBDS between markers of interest (in this case HLA and CD63) to the SBDS with a marker known to be diffusely expressed on all cells of interest (in this case CD45) permits SBDS interpretation (30). Quantification of fluorescent foci using is achieved using the “Spot Count” feature. A Threshold mask with adaptive erosion coefficient of 70 was applied to Channel 11 (HLA-A3) and served as the input for the “Spot Count” feature. This approach offered optimal spot identification sensitivity as confirmed by visual interrogation (although “Spot” masks or the “Spot Wizard” would be recommended in instances where spots might be less readily differentiated from background or for inexperienced users—see Figure S3 in Supplementary Material).

### Statistical Analysis

Unless otherwise stated, statistical analyses were performed by GraphPad Prism v7.0 Software. Student’s *t*-test was used for comparisons between two groups and ANOVA to compare more than two groups (*P < 0.05, **P < 0.01, ***P < 0.001, and ****P < 0.0001).

## Results

### Submicron Particle Detection and Multiparametric Characterization of Circulating sEVs

**Figure 1:**
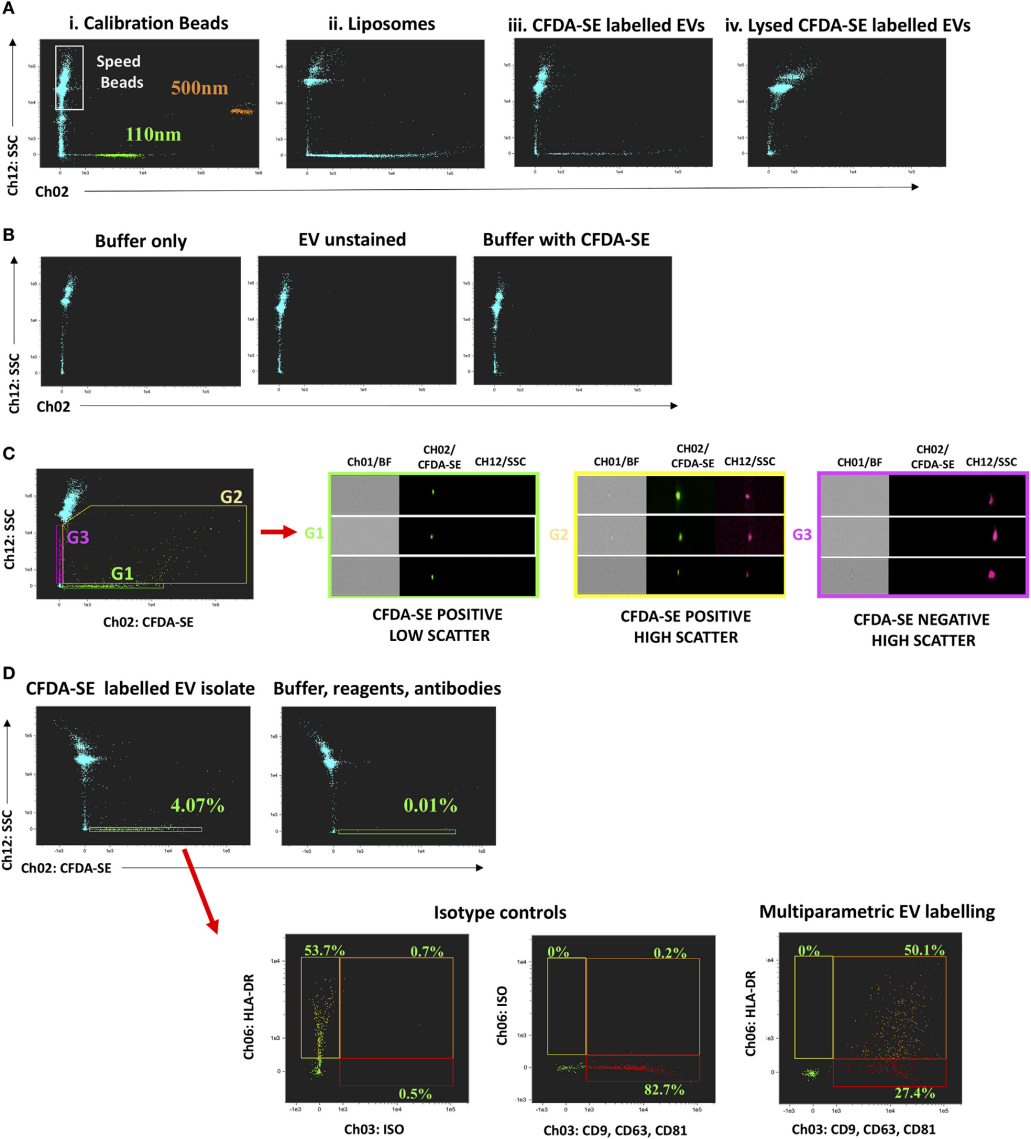
Submicron particle detection and multiparametric characterization of circulating “small EV” (sEV) by ImageStream^x^ (ISx). Gating sEV populations is achieved by assessment of the Scatter intensity of particles of known size, and through confirmation by single-event visual interrogation. **(A)** Fluorescently labeled polystyrene beads and liposomes which have a refractive index closer to that of EV are used and contrasted to CFDA-SE labeled circulating human extracellular vesicles (EVs) derived by ultracentrifugation. CFDA-SE-mediated labeling of intact vesicles is demonstrated by detergent lysis (0.1% Triton™ X-100) of UC-derived EVs prior to labeling. **(B)** The acquisition of appropriate control samples is an important step prior to running experimental samples if the common pitfalls of flow-cytometric profiling of EVs are to be avoided. Representative ISx dot-plots of buffer alone (filtered phosphate-buffered saline), unstained EVs, and buffer plus reagents without EVs are shown. **(C)** Representative dot-plot of CFDA-SE Vs. Scatter intensity to demonstrate the heterogeneity of acquired events and the principle of visual interrogation in gate-setting. The difference in properties of the two CFDA-SE-positive populations (G1 and G2) is clear by visual interrogation, showing G2 to be predominantly composed of cellular debris or particle aggregates as opposed to the uniform particles in G1—the sEV gate. **(D)** Multiparametric phenotyping of these gated sEV (G1) is demonstrated by fluorescent labeling with EV markers, the tetraspanins CD9, CD63, and CD81 combined (PE, Ch03) and HLA-DR (PECy7, Ch06). Fluorescence minus one and isotype controls are used to set gating. A buffer + reagent + antibody control should be performed and is demonstrated here.

The known capacity for submicron particle detection by iFCM was confirmed using fluorescent PSB 110 and 500 nm in size, with those of smallest size being readily resolved (Figure 1Ai). Manufactured beads are known to have higher refractive index (RI) than do biological vesicles of equivalent size (18, 31, 32). For this reason, gating strategy was also guided using fluorescently labeled liposomes of exosomal size (129.3 ± 2.4 nm) (Figure 1Aii; Figure S3B in Supplementary Material), which more closely resemble the RI and scatter characteristics of CFDA-SE-labeled sEVs (Figure 1Aiii). CFDA-SE serves as an intravesicular dye, becoming converted to CFSE once cleaved of acetate groups by esterases present in sEVs. Following detergent lysis of sEV isolates (0.1% Triton™ X-100), CFDA-SE labeling does not result in the detection of CFDA-SE-positive events, thereby confirming the requirement for vesicles to be intact for labeling to occur (Figure 1Aiv). The acquisition of appropriate control samples must be performed prior to each acquisition run, if common pitfalls associated with EV flow-cytometric profiling are to be avoided. Namely, this will help to avoid running contaminated reagents or antibodies which can lead to the acquisition of false particles. Representative ISx dot-plots of buffer alone (filtered phosphate-buffered saline), unstained EVs, and buffer plus reagents without EVs are shown (Figure 1B) and serve to reassure that none have been contaminated or formed aggregates for instance. In addition to the assessment of the scatter characteristics of fluorescently labeled particles of known size, gating of sEV populations is confirmed by single-event visual interrogation. While CFDA-SE-labeled sEVs appear as low-scatter and low/mid fluorescence intensity events, contaminating cells, EV clusters, and large EVs appear as higher scatter/intensity (14, 17, 18, 33). The difference in properties of the two CFDA-SE-positive populations (G1 and G2) is clear by visual interrogation of detected events, showing G2 to be predominantly composed of cellular debris or particle aggregates as opposed to the uniform particles in G1—the “small EV” gate (Figure 1C) (18). Multiparametric phenotyping of gated sEV (G1) was demonstrated by fluorescent labeling with EV markers, i.e., the tetraspanins CD9, CD63, CD81 (PE, Ch03), and HLA-DR (PECy7, Ch06). HLA-DR was chosen primarily for its ubiquity (Figure 1D). Appropriate isotype controls and FMO controls should be used for the setting of gates and interpretation of data. The importance of testing buffer with all antibodies and reagents to ensure that fluorescent particles with similar scatter to EVs are not produced leading to false events in G1 cannot be overstated (Figure 1D; Figure S4 in Supplementary Material).

### sEV Subtype Analysis and the Impact of Exosome Isolation Technique: Application 1

**Figure 2:**
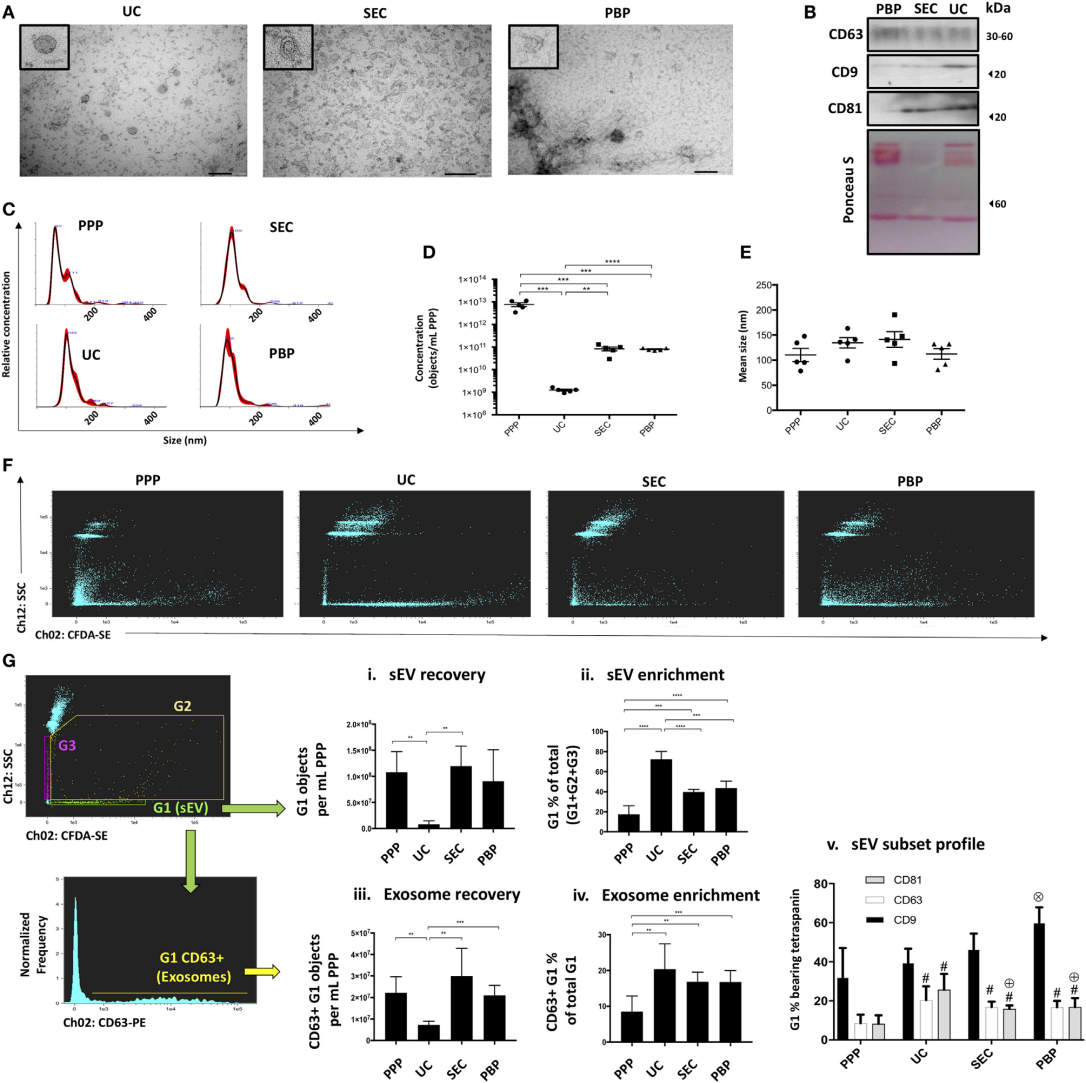
Extracellular vesicle subtype analysis and the impact of exosome isolation technique (Application 1). Confirmation of the presence of exosomes in “small EV” (sEV) isolates from three commonly used isolation techniques (UC, ultracentrifugation; SEC, size-exclusion chromatography; PBP, polymer-based precipitation) was achieved using three methods. **(A)** Transmission electron microscopy images showing characteristic morphologic features of exosomes (size bars: 200 nm). Single extracellular vesicles (EVs) are shown in expanded (zoomed) box in top left of each image. **(B)** Western blot showing canonical exosomal markers, such as CD9, CD63, and CD81. **(C–E)** Nanoparticle tracking analysis (NTA) profiling of isolates and of platelet poor plasma (PPP) showing size distribution and concentration profiles given “per mL of starting PPP” following correction for dilution/concentration steps were performed. **(F)** ISx dot-plot showing Scatter and CFDA-SE intensity for all methods of isolation. **(G)** ISx analysis of sEV subset recovery, including bona fide, CD63-positive, and exosomes. (i) sEV recovery is estimated by applying the objects per mL statistical feature in IDEAS™ to the G1 gate. (ii) The proportion of sEVs (G1) in relation to non-speed-bead events (G2 + G3) is used as an indication of sEV enrichment achieved by UC, SEC, and polymer-based precipitation (PBP) and compared to PPP. (iii) Exosome recovery is estimated by applying the objects per mL statistical feature in IDEAS™ to the CD63+ events within the G1 gate. In the cases of UC, SEC, and PBP, objects per mL relate to starting PPP sample, which is to say that the raw readout from the ISx “objects per mL” feature is corrected for any dilution/concentration steps performed. (iv) The proportion of exosomes (G1-gated CD63+ events) in relation to non-speed-bead events (G2 + G3) is used as an indication of exosome-specific enrichment achieved by UC, SEC, and PBP and compared to PPP. (v) Profiling of other canonical exosome markers CD9 and CD81, in comparison to bona fide exosome marker CD63. For consistency, representative data and images in panels **(A–C,F)** are from a single healthy volunteer, with panel **(G)** representative plots being UC-derived. Data represented as mean ± SEM of five healthy volunteers. *P < 0.05, **P < 0.01, ***P < 0.001, and ****P < 0.0001. ^#^*P* < 0.05 relative to PPP. ^⊕^*P* < 0.05 relative to UC. ^⊗^*P* < 0.05 relative to PPP, UC, and SEC.

To demonstrate the capacity and utility of iFCM in sEV subtype analysis, isolates from three commonly used “exosome” isolation techniques were compared. The successful isolation of exosomes from the plasma of five healthy volunteers by three commonly utilized techniques—ultracentrifugation (UC), SEC, and PBP—was confirmed by TEM, NTA, and Western blot for canonical exosomal markers (Figures 2A–E; Figure S5 in Supplementary Material). The size profile of isolated vesicles, as determined by NTA, was equivalent across isolation approaches. Estimation of the concentration of sEVs by NTA shows UC to isolate significantly fewer particles per mL plasma than SEC or PBP (Figure 2D) in keeping with previous reports (22, 34, 35).

Analysis of the Scatter/CFDA-SE profiles of samples revealed that as a percentage of all detected non-speed bead events sEVs were lowest in PPP, thereby confirming successful sEV enrichment by all isolation methods tested (Figures 2F,Gii). The greatest enrichment or “purification” of sEV was seen in UC isolated samples (Figures 2F,Gii). However, the recovery of sEVs from plasma was significantly less in the case of UC compared to other methods, as quantified by the “objects per mL” function (Figure 2Gi). PPP was noted to contain relatively high proportions of CFDA-SE-negative particles displaying varying degrees of Scatter (G3), but also of CFDA-SE-positive particles with high Scatter than sEVs (G2), likely to represent debris, EV clusters, large EVs, or apoptotic bodies. SEC and PBP samples also exhibited relatively higher proportions of high-Scatter events, thereby contributing to their lower sEV enrichment estimation as compared to UC (Figures 2F,G). sEV subset analysis was performed by assessing the number and proportions of gated sEVs (G1) bearing subset markers. Again, all three methods achieved *enrichment* from PPP starting material of bona fide exosomes—defined as G1-gated CD63+ events (Figure 2Giv). However, the *recovery* of bona fide exosomes, assessed as the number of CD63 + sEV “objects per mL”, was also shown to be lowest following UC (Figure 2Giii). CD9 was observed to be the most commonly contained tetraspanin in sEVs across all samples, while CD81 was noted to be particularly enriched in UC isolates (Figure 2Gv). Taken together, iFCM analysis confirmed that the recovery and the purity of sEV subsets, as defined by their content of subset-specific markers, vary among isolation techniques.

### Profiling of Circulating Exosomes for Tissue-Specific Biomarker Discovery and Functional Analysis: Application 2

The utility of exosomes as biomarkers has been extolled due to their stability and ubiquity in biofluids, their capacity to offer insights into the cell or compartment of origin, and for their selectively enriched cargo which offers an understanding of their functional impact upon cellular targets. For these reasons, a convenient, sensitive, and robust approach to their characterization from circulating blood would be of significant benefit.

To demonstrate circulating exosome detection and characterization by iFCM, the setting of clinical organ transplantation offered an optimal scenario. It provided the opportunity to combine the use of an exosome-specific marker (CD63), with an origin-specific marker (donor-HLA). In addition, the setting enabled the detection of a relatively rare population of tissue-specific exosomes with the same patient serving as the control subject (pre-transplantation) so as to rule out non-specific antibody targeting. Finally, additional markers allowed functional assessments and comparisons to be made between donor- and recipient-origin exosomes.

**Figure 3:**
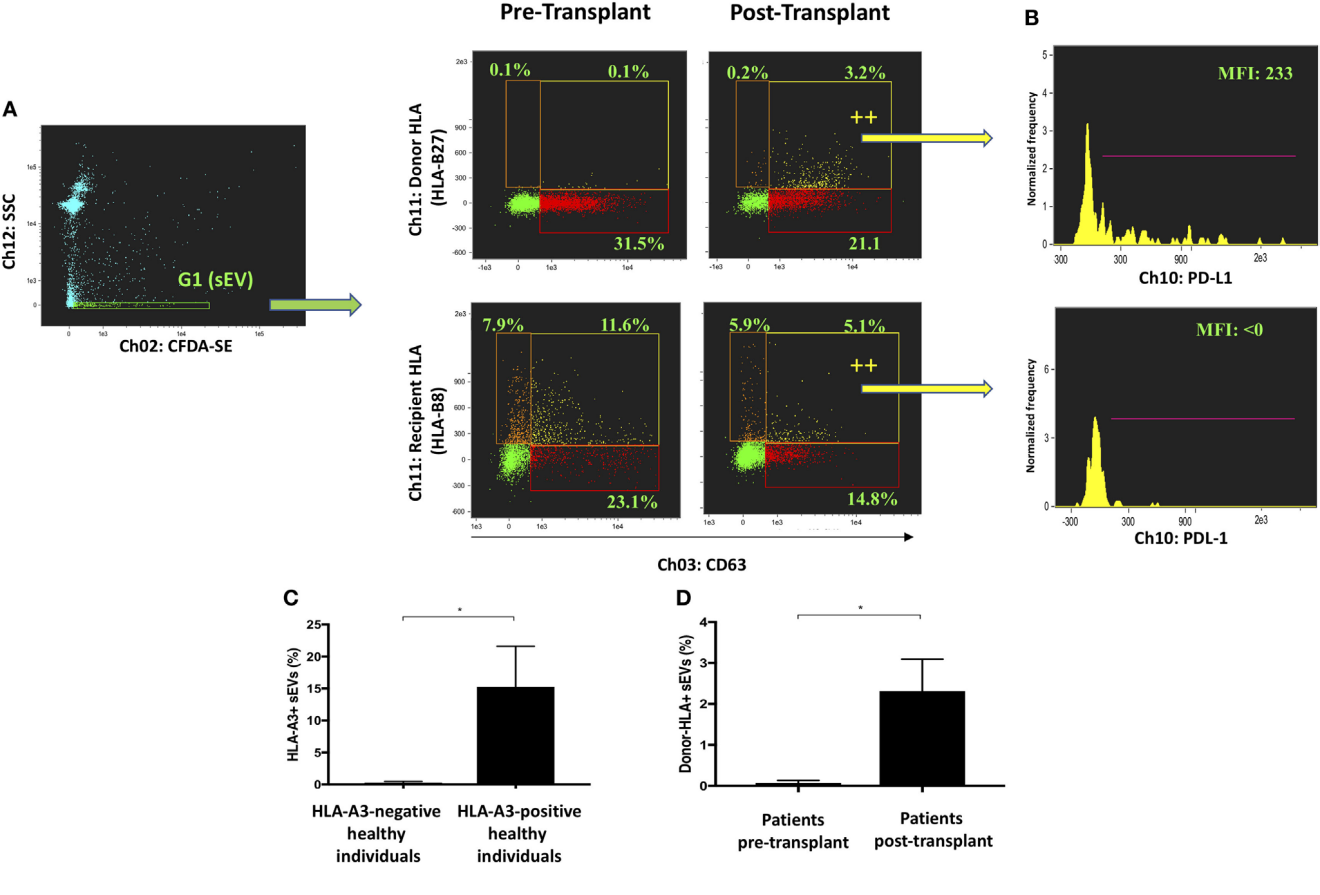
Profiling of circulating exosomes for tissue-specific biomarker discovery and functional analysis (Application 2). **(A)** Small EVs (sEVs) from an HLA-B27-positive, HLA-B8-negative recipient were gated in G1 before and after receiving a liver allograft from an HLA-B8-positive, HLA-B27-negative donor. Bona fide CD63-positive exosomes were analyzed for their expression of donor and recipient HLA. Donor HLA-bearing exosomes become detectable in circulation after liver transplantation. **(B)** PD-L1 expression was analyzed in exosomes bearing either donor or recipient HLA and found higher in the former group post-transplantation. **(C)** Percentage of HLA-A3 positive sEVs were compared between genotypically confirmed HLA-A3+ (*n* = 3) and HLA-A3 negative (*n* = 3) individuals. **(D)** Percentage of donor-HLA + sEVs observed pre- and post-liver transplantation, in genotypically confirmed HLA-mismatched liver transplant recipients (*n*3). Data represented as mean ± SD. *P < 0.05.

Bona fide CD63-positive exosomes were analyzed for their expression of donor and recipient HLA (Figure 3A). sEVs from an HLA-B27-positive, HLA-B8-negative recipient were gated in G1 before and after receiving a liver allograft from an HLA-B8-positive, HLA-B27-negative donor. Donor HLA-bearing exosomes were noted to be detectable in circulation after liver transplantation. These results were further substantiated by comparing healthy volunteers of distinct HLA genotype, as well as additional liver transplant recipients (Figures 3C,D). Multiparametric iFCM analysis enabled functional comparisons of exosome populations. It has been suggested that programmed death-ligand 1 (PD-L1) released from allografts in a mouse model of liver transplantation are responsible for T cell exhaustion and tolerogenesis (36). Therefore, we sought to assess the differential distribution of PD-L1 in circulating exosomes of transplanted patients. Interestingly, the presence of PD-L1 was seen to be higher on donor-origin liver-derived exosomes (Figure 3B). This approach to circulating plasma-derived exosome characterization permits convenient origin identification and functional assessment to be performed in a variety of contexts.

### Target Cell Analysis Determines Exosome-Mediated Molecular Transfer: Application 3

**Figure 4:**
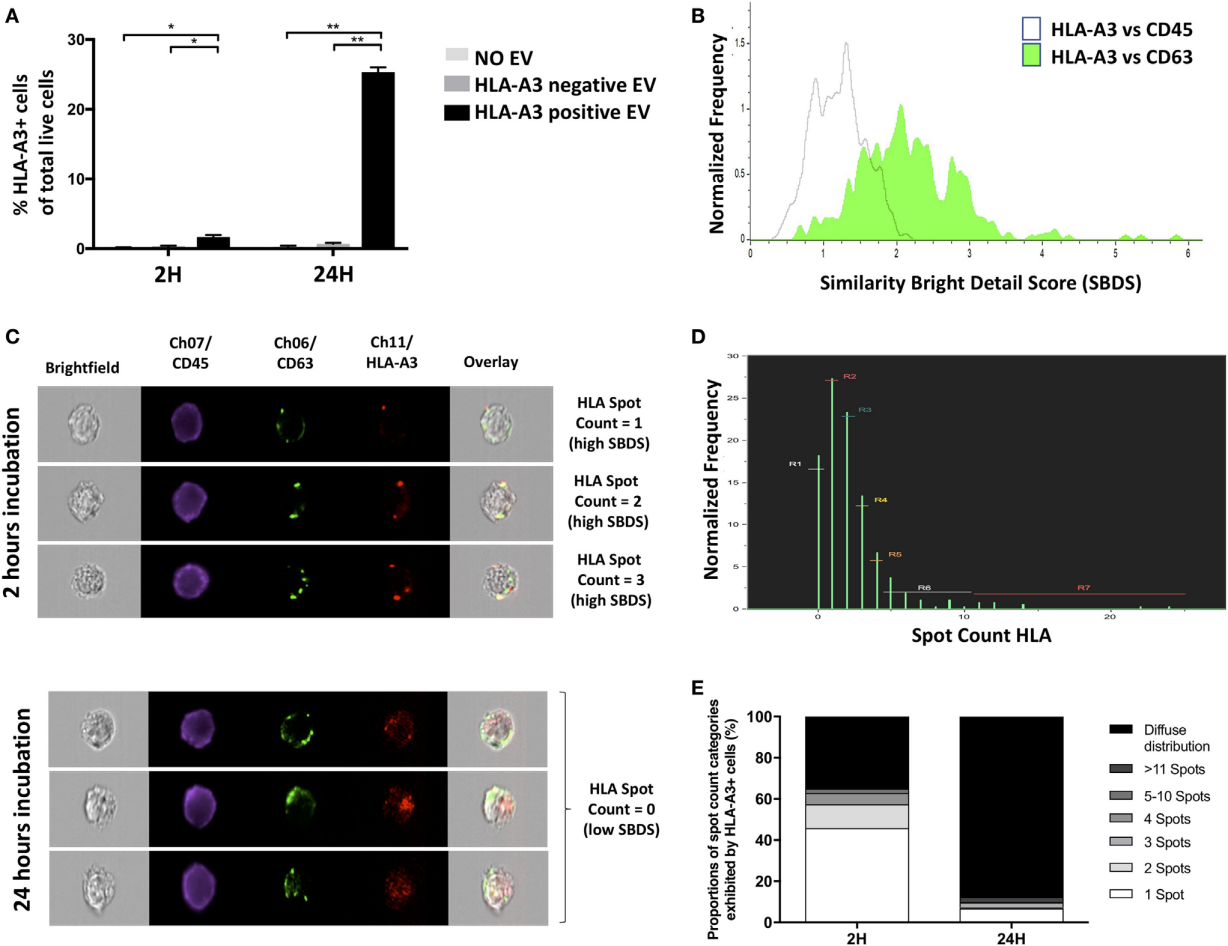
Target-cell analysis determines exosome-mediated molecular transfer (Application 3). **(A)** Percentage of HLA-A3-positive cells detected by ISx following coculture of CD14+ cells from HLA-A3-negative individual with extracellular vesicles from HLA-A3 positive individual. Data represented as mean ± SD of two separate experiments. *P < 0.05 and **P < 0.01. **(B)** Representative histogram showing higher colocalization of fluorescently labeled HLA-A3 with CD63 as compared to CD45, as ascertained by the IDEAS^®^ Similarity Bright Detail Score (SBDS). **(C)** Representative ISx images of single cells following 2 h coculture exhibiting discrete HLA-A3 spots and with high HLA-A3/CD63 SBDS scores (left panels), contrasted with images of cells following 24 h coculture exhibiting diffuse HLA-A3 fluorescence and with low HLA-A3/CD63 SBDS scores; suggesting incorporation and diffusion of exosome-derived HLA molecules over time. **(D)** Representative histogram showing the normalized frequencies of HLA-A3 positive cells exhibiting a given number of HLA-A3 “spots,” as ascertained by the IDEAS^®^ Spot Count feature, following 2 h EV coculture. **(E)** Summary of proportions of HLA-A3 positive cells exhibiting a given number of HLA-A3 spots, following either 2 or 24 h EV coculture. Data presented as the mean of two separate experiments.

Establishing definitively whether molecules found on a cell’s surface were exosomally transferred is difficult in clinical contexts where pre-labeling of exosomes is not possible. Exosome-mediated transfer might, however, be inferred by assessing whether the molecule of interest is colocalized with exosome-specific proteins such as CD63, since at the time of incorporation to the target cell the exosome leaves a patch of the proteins it transferred. To this end, monocytes from an HLA-A3 negative individual were incubated *in vitro* with sEVs from HLA-A3 positive and HLA-A3 negative (control) individuals. The colocalization of exosomal marker CD63 with HLA-A3 was assessed using the Bright Detail Similarity Score (SBDS) within IDEAS^®^, as described previously. CD63 was chosen because it defines bona fide exosomes, but also because in most cells it is present predominantly in intracellular compartments rather than the cell surface (37). Following 2 h incubation of monocytes with sEVs derived from an HLA-A3 donor, the proportion of HLA-A3 positive monocytes increased significantly and continued to rise to 24 h (Figure 4A). SBDS for HLA-A3 and CD63 colocalization was significantly higher than among the CD45/CD63 comparator, with CD45 (“leukocyte common antigen”) serving as a total-surface stain (SBDS 2.26 ± 0.21 Vs. 1.12 ± 0.11; *P* < 0.01; Figure 4B). HLA-A3 fluorescence was confirmed to colocalize with CD63 by visual interrogation of high SBDS events (Figure 4C). It should be noted that not all HLA-A3 “spots” were associated with CD63; likely a reflection of our data showing that not all sEVs are CD63 containing. Following 24 h incubation, and without further addition of sEVs, ISx image interrogation also provided clues as to the kinetics of HLA/exosome uptake. At this time point, HLA-A3 positive monocytes were either studded with a *multitude* of HLA-A3 foci, and/or exhibited diffuse fluorescence in the HLA-A3 channel (Figure 4C). These kinetic properties could be quantitated using the “spot count” feature in IDEAS^®^, as described above, with data showing clear trends toward either no spots (diffuse fluorescence) or multiple spots (>11) among HLA-A3 positive monocytes undergoing a longer incubation period (Figures 4D,E). So, spatial interrogation of acquired cells using ISx and IDEAS^®^ reveals that exosome-mediated transfer of allo-HLA to CD14+ cells occurs rapidly, with HLA initially limited to the area of exosome binding followed by more generalized dispersal.

## Discussion

In the present report, we address the need for a convenient method by which high-throughput multiparametric phenotypic and functional analyses of sEV subsets, in particular exosomes, can be performed.

The superiority of iFCM over cFCM in the detection of submicron particles has been previously described (14, 18). ISx utilization of spatially registered CCD cameras, rather than photomultiplier tubes, allows for superior fluorophore detection sensitivity. While machine noise plagues cFCM sensitivity and necessitates a triggering threshold to be set, the ISx CCD does not have to exceed a threshold value above which a particle can be considered a data point. Instead, it acquires all images that are at least one pixel above the background of the camera (14, 17, 38). The ISx calibration software, run upon every startup, performs pixel gain corrections and dark current offsetting for each one of the CCDs pixels, thereby further enhancing the detection of faint signals and providing a uniform background level (38). In practical terms, the result is that ISx is able to detect and resolve 20 nm fluorescent latex beads which are entirely undetectable by cFCM (17). Importantly, unlike cFCM, ISx also suffers less from swarm, with coincident detection leading to significant underestimation of counted populations. Finally, unlike many reports of EV characterization by cFCM, there is also no requirement for specially adapted cytometers or an experienced operator capable of manual hardware adjustments and calibrations (24, 39). “Dedicated” small particle cytometry (dFCM) platforms such as these meet many of the requirements for sEV analysis, including high powered lasers, a stable velocity core stream, and higher sensitivity detectors (40). However, to date, they do not offer the convenience of use required by many researchers. Furthermore, cFCM (and dFCM) is low in information content, measuring only one feature per fluorescence marker (integrated intensity); compared to iFCM which allows thousands of spatial and morphological properties of individual events to be assessed—thereby permitting analyses such as those outlined in “Application 3.”

Drawing on their comprehensive proteomic analyses of EVs derived from human dendritic cells, Théry and colleagues set out a sub-classification of sEVs according to tetraspanin markers (7). Different approaches to exosome isolation are known to skew the recovery of subsets toward more or fewer vesicles bearing one or another of these markers, and so the functional properties of isolates derived by different techniques are known to vary (7, 22, 41). Indeed, this might go some way toward explaining apparent contradictions reported across published studies of EV functional analyses (2, 7). That is to say, since variations exist in isolation procedures among groups, it must be expected that the functional properties of isolates also vary. Such “skewing” of EV isolate is not only restricted to the specific isolation technique used, with variations in the steps involved in the preparation of plasma also having an impact. These range from venepuncture technique, the choice of blood collection tube, the length of incubation time, the means of transportation, the storage temperature, the centrifugation approach, and the number of freeze thaw cycles, to name but a few (8, 42). Of course, much emphasis has been given to the need for standardization of EV isolation (19). But, it comes as little surprise, that calls have been made by researchers leading the field for improved techniques enabling the accurate characterization of different populations of sEVs, including exosomes (2, 10, 43). We show how iFCM analysis of sEV isolates offers a convenient, accurate means by which this can be achieved.

In our analysis we confirm that commonly used “exosome” isolation techniques produce differing isolates. The focus of the interpretation of this effect is not so much on making a suggestion about the relative superiority/inferiority of a particular isolation method. As mentioned, the exact outcomes of such analysis would likely vary with small alterations in protocol, between different investigated patients, across the spectrum of biofluids, and so on. Rather, it is to be reminded that this variation exists, and to be mindful of the need for subset characterization in a number of contexts. We suggest iFCM is a convenient way to achieve this also. Examples of the instances where sEV subtype profiling may be needed include: (a) to ensure consistency of isolation from a given sample, and so achieve consistency in any analyses performed thereafter; (b) to assist standardization of isolation across different researchers, permitting collaboration or replication; (c) enable interpretations of downstream isolate analysis in terms of the subtype profile; and (d) to provide *quantitative* confirmation of the presence of exosomes in conjunction with current methods (TEM, Western, NTA). In addition, such analysis could guide the choice of method most appropriate for the requirements of selected downstream analyses. For instance, our data might suggest that if a limited volume of plasma is available and exosomal miRNA profiling is desired, UC may not be the best option due to the low quantity of exosomes recovered. If a larger volume of plasma is available, however, UC may be preferred for the greater enrichment of exosomes achieved. Of course, considerations such as the co-isolation of proteins are important in this context.

Numerous studies purporting to be investigating circulating exosomes, performed predominantly in the pursuit of biomarkers of disease, continue to apply analytic techniques ill-suited to the study of exosomes. The use of flow cytometry platforms incapable of accurately resolving objects of exosomal size is one concern, while the failure to narrow investigations specifically to exosomes through the use of appropriate markers is another. An assumption that the “exosome” isolation technique employed *purifies* exosomes, can lead to erroneous conclusions regarding what in fact is the analysis of a heterogeneous pool of sEV of divergent functional properties. We demonstrate that iFCM coupled with subset-specific markers can be applied to circulating, plasma-derived exosome characterization in order to achieve reliable exosomal-origin determination and functional assessments. Multiparametric analysis can provide insights into the immune function of exosomes identified in this way. This approach should be applicable to the gamut of biofluids, across species, or with a focus on any particular sEV subtype. Due to sEVs’ small size, however, the question of whether steric hindrance may affect the number of different antibodies that can be used in labeling remains to be investigated, and so the design of large panels should be approached with caution and will require extensive validation. We have shown that “unprocessed” samples (PPP) can also be used, thereby circumventing adverse impacts of isolation procedures. However, the ISx fluidics requires that small sample volumes are run, and that these are run slowly. Thus, samples need to be sufficiently concentrated if long acquisition times are to be avoided.

The fluidic control of ISx coupled with the presence of continuously running speed beads enable cell/particle enumeration using the “objects per mL” feature within the IDEAS^®^. Our data show this feature to corroborate NTA data in comparisons of the relative concentrations of EVs present in isolates derived by different methods. However, the two approaches were very different in the absolute number of EVs by some orders of magnitude. Enumeration of EVs is notoriously challenging and has yielded widely divergent results (44). A number of platforms are currently in use for EV enumeration, with NTA being among the most commonly applied. Each method has its own merits and drawback, and NTA also suffers from the latter. The device is unable to distinguish vesicles from non-vesicular “debris” which by ISx would be identified and excluded by its CFDA-SE-negativity. Inter-user and inter-experiment variability is also a known concern with NTA, particularly among inexperienced users, though steps are being taken to improve this (45–48). The merits of FCM for particle enumeration over other techniques such as NTA are beyond our scope and described elsewhere (14, 49, 50). What is noteworthy however, is the capacity for iFCM to enumerate sEV *subsets* specifically, rather than vesicles taken together, or indeed merely the vesicle-sized particles measured by some other techniques. More work is needed for a standardized approach to particle enumeration to be agreed upon.

The use of ISx is also associated with some drawbacks. The particularly large file sizes produced require appropriate storage and processing capacity on any computers used for data analysis, and analyzing data be extremely time intensive. Each alteration to a compensation matrix once acquisition has been performed, requires large files to be re-processed and re-opened. The merging of large files for merged graphical analyses is also laborious. While the setting up of Masks and Feature functions requires the development of some familiarity with the analysis software, the provided Wizards can greatly assist in this process and once satisfactory set-up is achieved, files can be easily batch processed.

## Conclusion

Taken together, the data and methods presented offer solutions to two important problems facing EV research. The first relates to the necessity for the subtype characterization of sEV isolates. Without this, the field will continue to produce apparently contradictory EV functional assessments, with comparison or collaboration between individuals or across centers remaining difficult. The second is the need for a validated approach for the multiparametric analysis of bona fide circulating exosomes. Here, we show this to be possible by coupling our emerging understanding of subset-specific markers with the use of advanced iFCM. In this synthesis of our experience characterizing sEVs, we hope to offer a reference to guide EV researchers, perhaps new to the field, in the necessary subset-analysis of isolates, in the multiparametric profiling of exosomes or other sEV subsets, and in the assessment of exosome-mediated molecular transfer. In doing so, we hope to support ongoing research into the function and clinical utility of EVs.

## Ethics Statement

The study was approved by the North of Scotland Research Ethics Committee (REC Ref: 15/NS/0062). All subjects gave written informed consent in accordance with the Declaration of Helsinki.

## Author Contributions

SM, GB, FD, AS-F, and MM-L designed the study and developed methodology. SM and GW collected samples. SM, GB, and MM performed and analyzed experiments. SM, MM-L, and AS-F prepared the manuscript. MM-L and AS-F supervised the project.

## Conflict of Interest Statement

The authors declare that the research was conducted in the absence of any commercial or financial relationships that could be construed as a potential conflict of interest.
