# Supplementary material for: Multiparametric Analysis of Circulating Exosomes and Other Small Extracellular Vesicles by Advanced Imaging Flow Cytometry
Source: Front Immunol. 2018 Jul 6;9:1583. doi: 10.3389/fimmu.2018.01583 (PMC6043651; doi:10.3389/fimmu.2018.01583)
Supplement: Supplementary file 1 [file presentation_1.PDF]

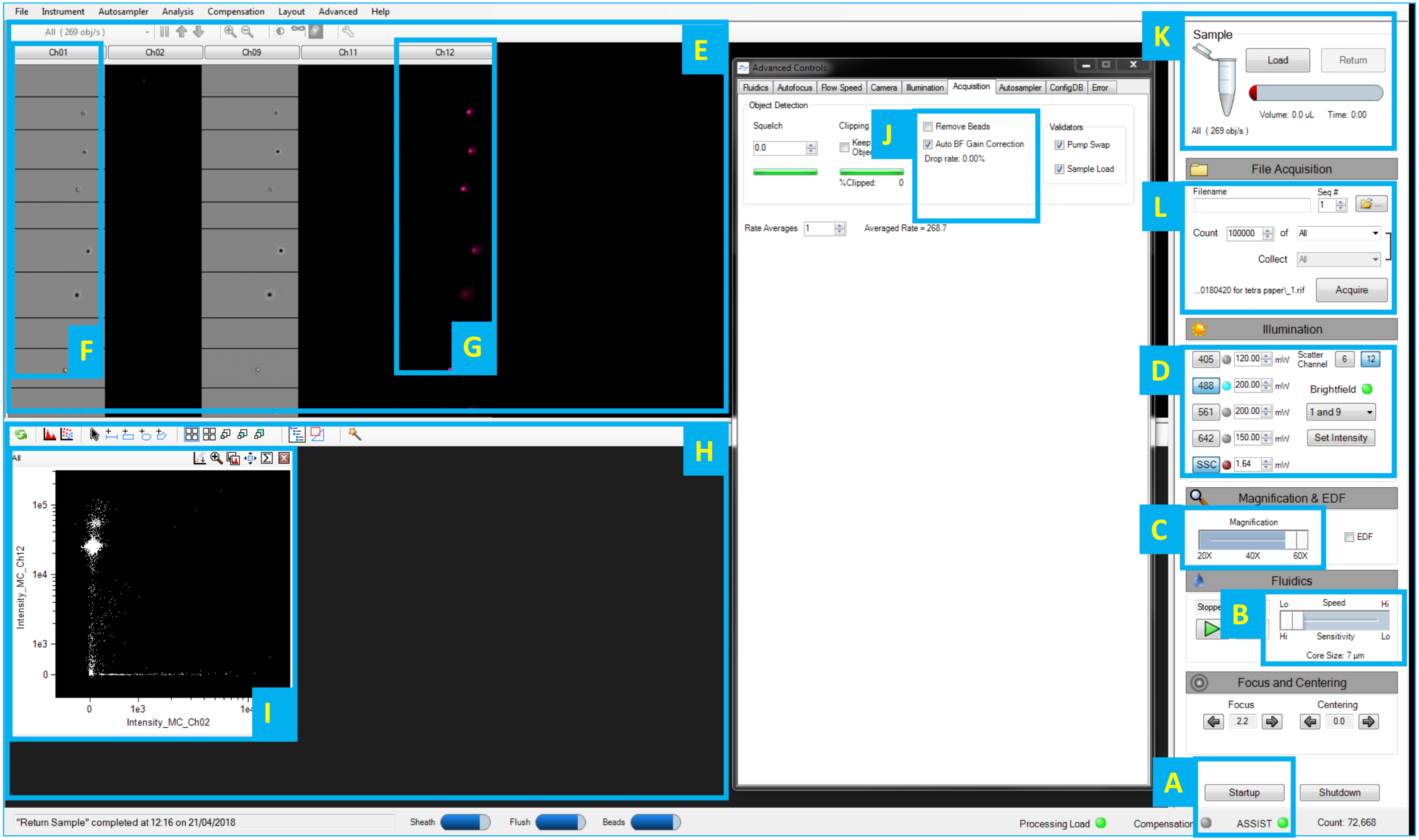

Supplementary Figure S1. INSPIRE® data acquisition software. ISx calibration software is run upon every startup **(A)**. Instrument controls **(B-D)** are set to the highest resolution and lowest running speeds, with 60x magnification selected, a Core Size of 7µm, and all lasers set to maximum power. Image gallery **(E)** enables real-time viewing of images of events being collected, and of each of the selected detection channels including brightfield **(F)** and Scatter **(G)**. Work area **(H)** permits real-time graphical gating using dot-plots **(I)** and other modalities. Following unchecking of 'Remove beads' which must be performed in the 'Advanced Controls' menu prior to each acquisition **(J)**, and the setting of collection gate and counting gate parameters **(L)**, acquisition can be started **(K)**. ISx possesses 12 spectral image detection channels (Ch01-Ch12): Ch01 420-480nm, Ch02 480-560nm, Ch03 560-595nm, Ch04 595-660nm, Ch05 660-740nm, Ch06 740-800nm, Ch07 420-505nm, Ch08 505-570nm, Ch09 570-595nm, Ch10 595-660nm, Ch11 660-740nm, Ch12 740-800nm. ISx was equipped with 5 excitation lasers: 405nm diode laser, 488nm solid state laser, 561nm solid state laser, 592nm solid state laser, and 658nm diode laser.

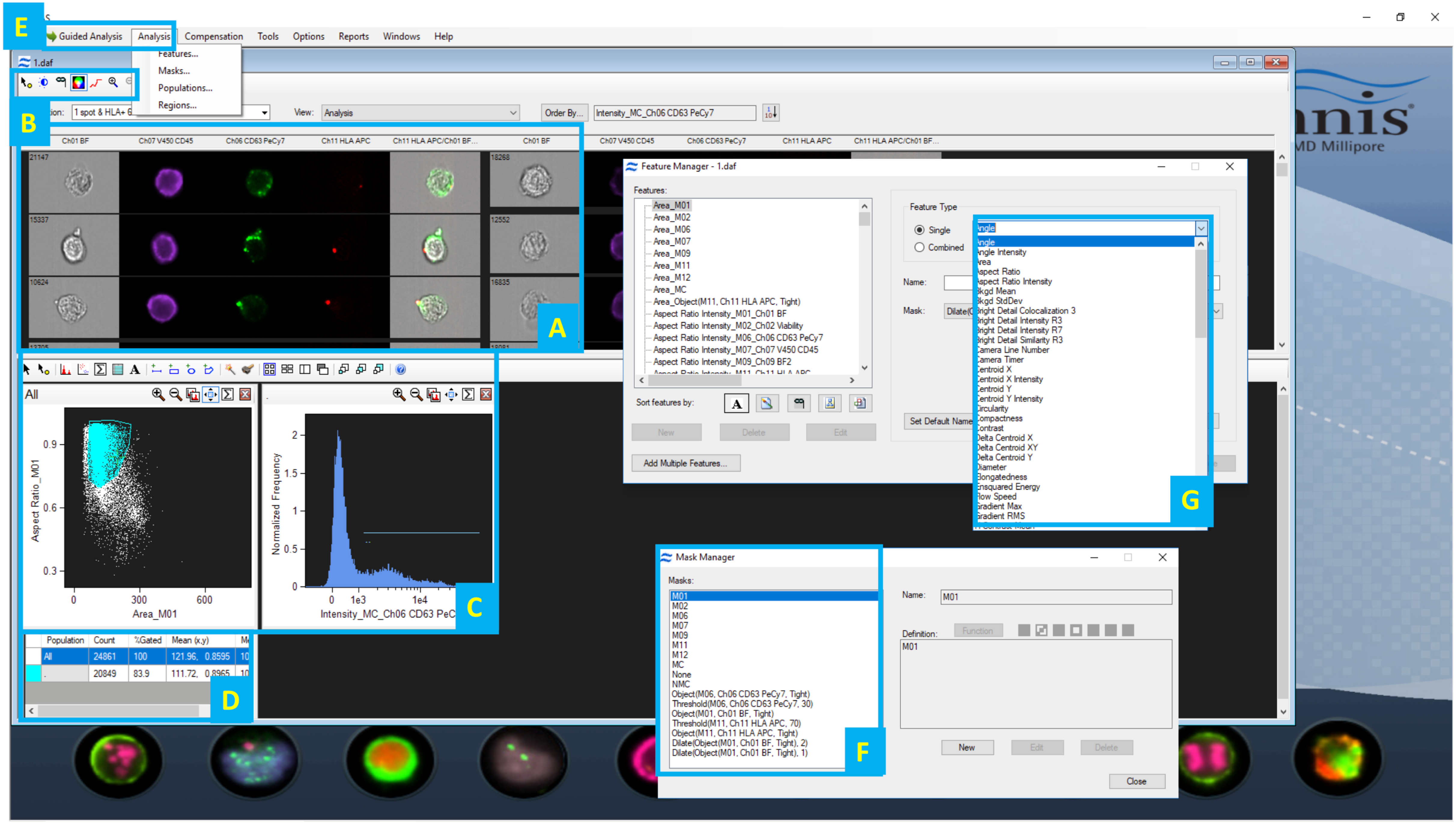

Supplementary Figure S2. IDEAS® data analysis software. The image gallery **(A)** allows visual inspection of selected cells, and can be customized to show particular channels, masks, and image overlays among other options **(B)**. Extensive graphical analysis tools are available in the work area **(C)**, and cells displayed in the image gallery can be selected through the definition of populations or by selecting specific dots to visualize single cells. Comprehensive population statistics can also be applied in this area **(D)**. Guided analyses using wizards are available for common applications **(E)**. Manual application of masks is achieved in the Mask Manager **(F)**, and further computation of masks and other parameters can be performed in the Features Manager **(G)**.

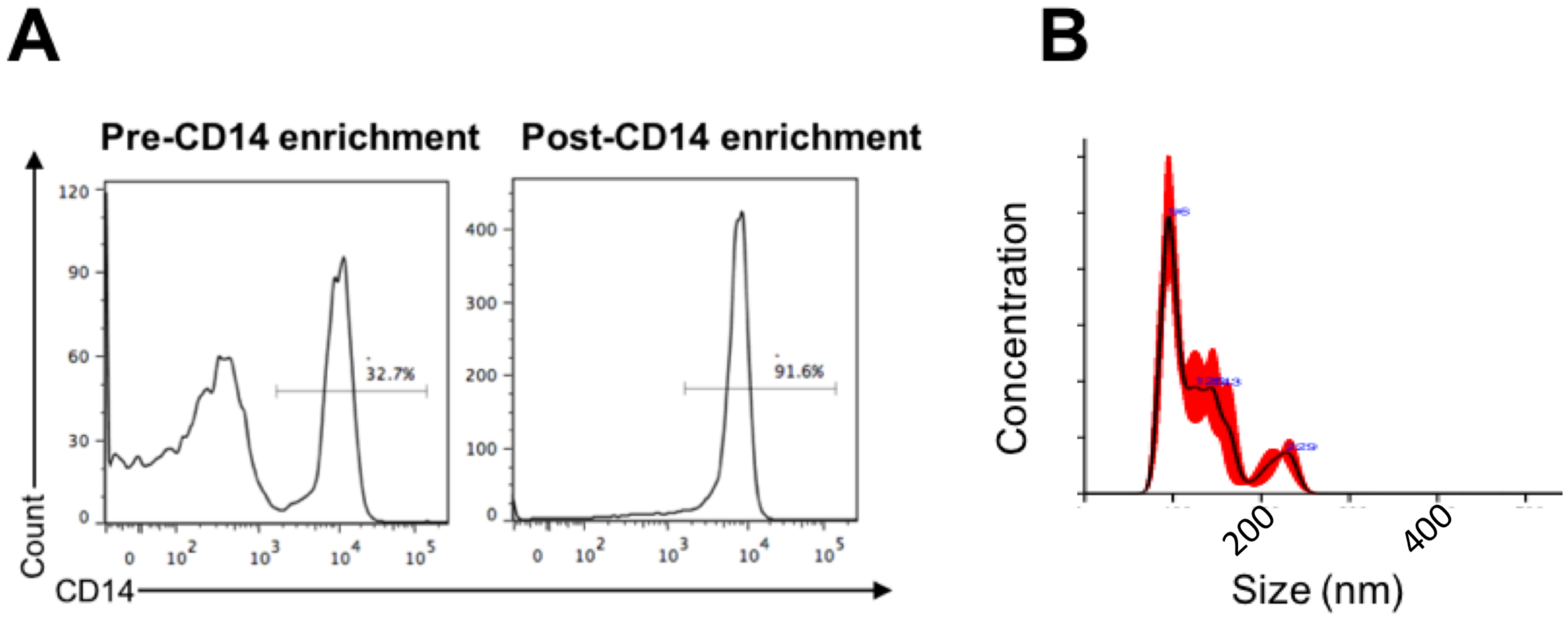

Supplementary Figure S3. (A) Representative flow cytometric confirmation of viable CD14+ monocyte enrichment following immunomagnetic bead isolation by EasySep™ (StemCell Technologies). Purities over >90% were achieved in all experimental procedures performed (CD14-PECy7, Biolegend; LIVE/DEAD™-APC, ThermoFisher Scientific). **(B)** Nanoparticle tracking analysis (NTA) showing size profile of CF-liposomes (Mean size 129.3+/-2.4nm).

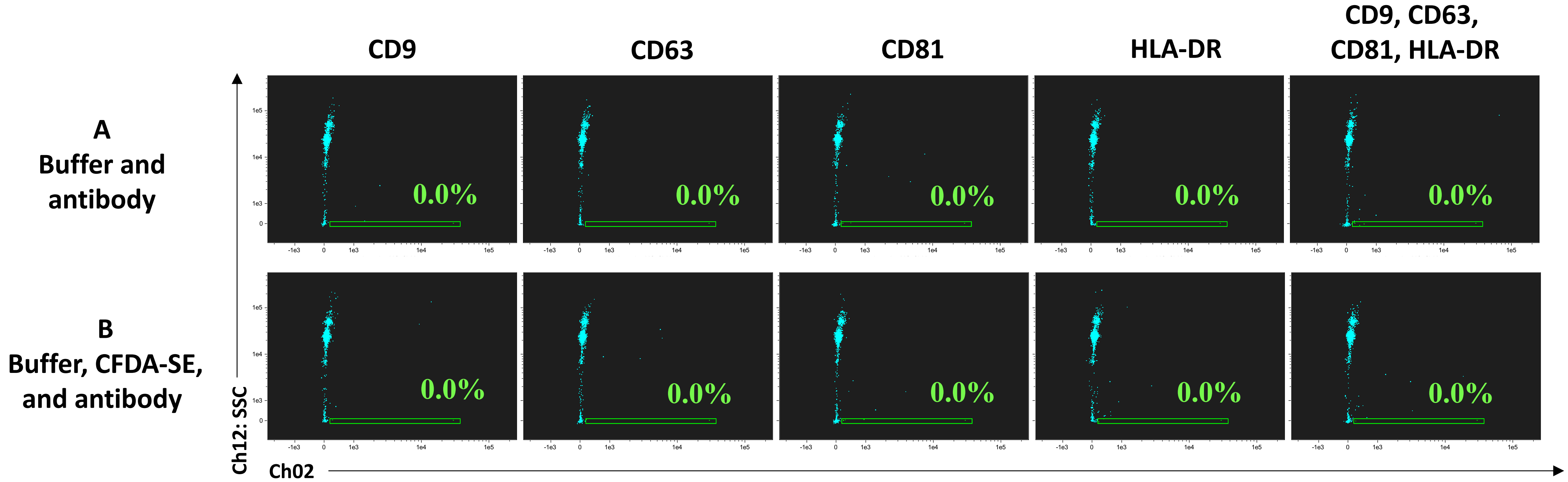

Supplementary Figure S4. A number of control samples must be run alongside each experiment. In Figure 1B we show ‘buffer only’ and ‘buffer with reagents’ controls; the latter containing CFDA-SE. In addition, Figure 1D shows the ‘buffer with all used antibodies and reagents’ control, which is important in establishing the absence of false positive events in G1. Here, for completeness, antibodies individually and in combination are run in buffer alone (upper row, A), or in buffer with CFDA-SE (lower row, B). Percentage of G1 gated events is shown in each case.

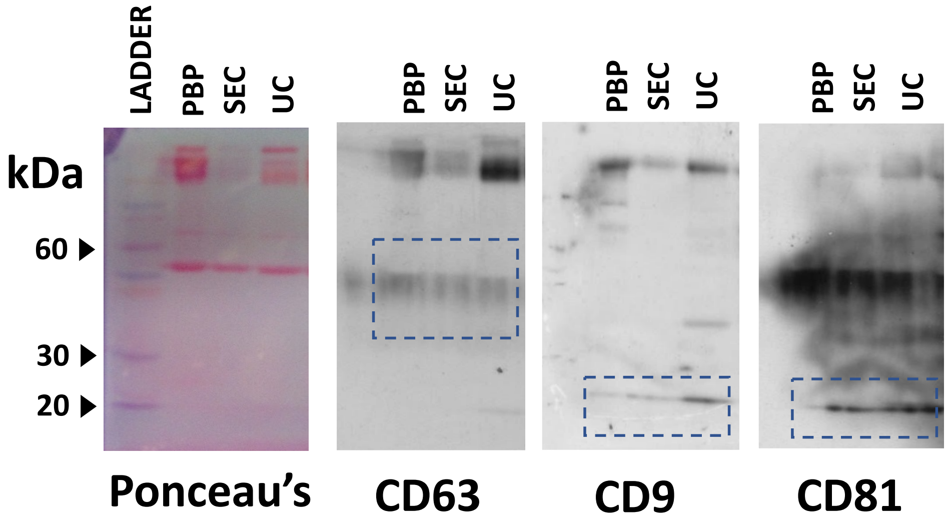

Supplementary Figure S5. Western blots confirming the presence of tetraspanins CD9, CD63, and CD81 in EVs isolated by three methods: polymer-based precipitation (PBP), size-exclusion chromatography (SEC), and ultracentrifugation (UC).
